# Supplementary material for: Ethmoid bone marrow region modulates lymphoid activation and differentiation with distinct myeloid profile in neuroinflammation
Source: bioRxiv. 2026 Oct 17:2026.10.03.755016. Preprint. [Version 3] doi: 10.64898/2026.10.03.755016 (PMC13692023; doi:10.64898/2026.10.03.755016)
Supplement: Supplementary file 1 [file NIHPP2026.10.03.755016V3-supplement-1.pdf]

bioRxiv preprint doi: https://doi.org/10.64898/2026.10.03.755016; this version posted October 17, 2026. The copyright holder for this preprint (which was not certified by peer review) is the author/funder, who has granted bioRxiv a license to display the preprint in perpetuity. It is made available under a CC-BY 4.0 International license.

Supplementary Figure 1

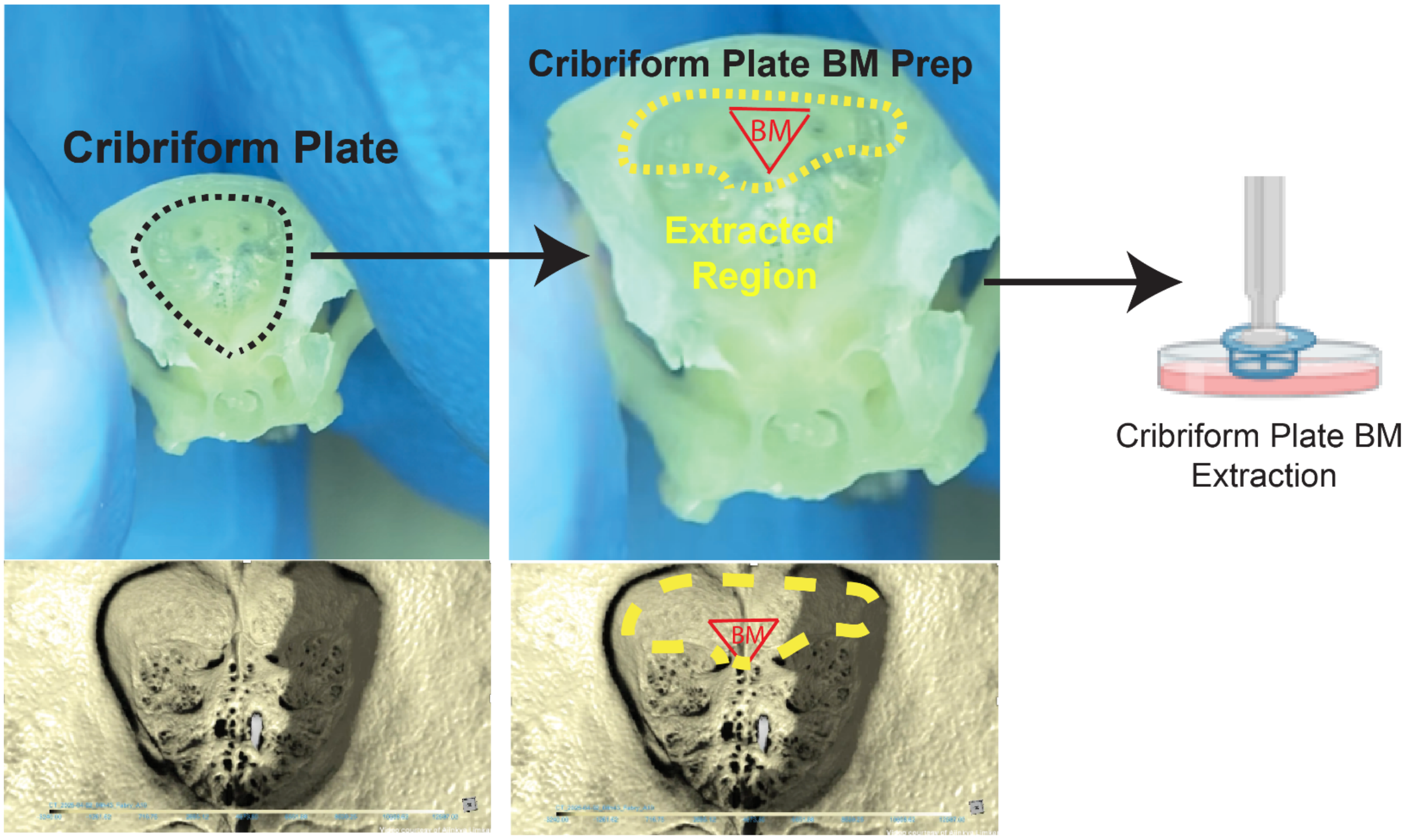

bioRxiv preprint doi: https://doi.org/10.64898/2026.10.03.755016; this version posted October 17, 2026. The copyright holder for this preprint (which was not certified by peer review) is the author/funder, who has granted bioRxiv a license to display the preprint in perpetuity. It is made available under a CC-BY 4.0 International license.

Supplementary Figure 1

Representative images showing isolated cpBM regions. Samples were washed with PBS prior to dissociation. Samples were enriched for cpBM but contained a broader cpBM region environment as well. CT image provided by Ajinkya R Limar and Sophia Vrba.

bioRxiv preprint doi: https://doi.org/10.64898/2026.10.03.755016; this version posted October 17, 2026. The copyright holder for this preprint (which was not certified by peer review) is the author/funder, who has granted bioRxiv a license to display the preprint in perpetuity. It is made available under a CC-BY 4.0 International license.

Supplementary Figure 2

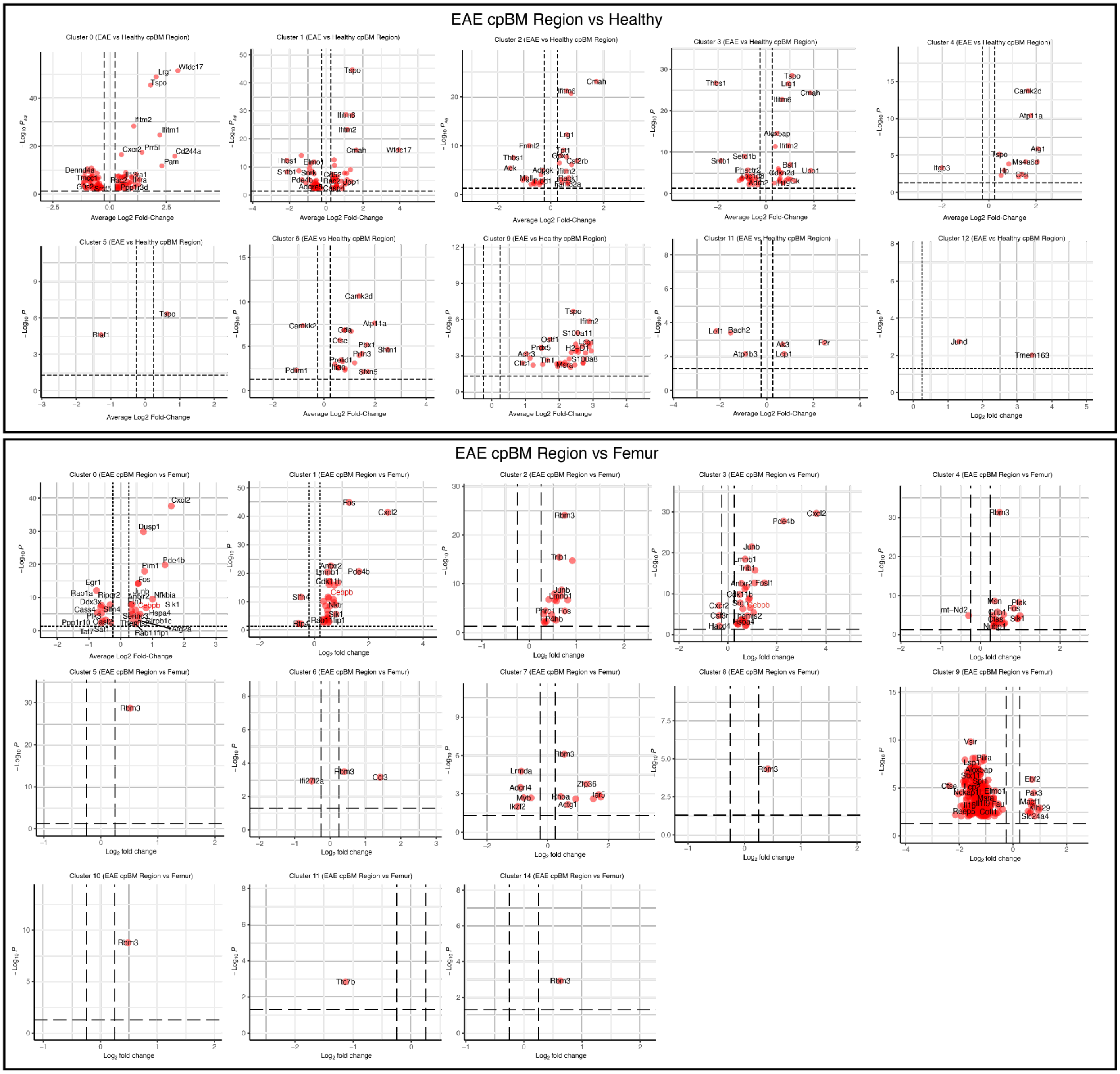

bioRxiv preprint doi: https://doi.org/10.64898/2026.10.03.755016; this version posted October 17, 2026. The copyright holder for this preprint (which was not certified by peer review) is the author/funder, who has granted bioRxiv a license to display the preprint in perpetuity. It is made available under a CC-BY 4.0 International license.

Supplementary Figure 2

Volcano plots for clusters with significant differences between EAE cpBM vs Healthy cpBM or EAE feumur BM.

Top plots show EAE vs Healthy cpBM comparisons, while bottom shows EAE cpBM region vs EAE femur.
